# The Core of Sibling Stem Cell Donation – A Grounded Theory Study

**DOI:** 10.2174/1874434601711010073

**Published:** 2017-06-30

**Authors:** Annika M Kisch, Anna Forsberg

**Affiliations:** 1 Department of Haematology, Skåne University hospital, Lund, Sweden; 2 Lund University, Inst of Health Sciences and Department of Thoracic Surgery, Skåne University Hospital, Lund, Sweden

**Keywords:** Stem cell donation, Sibling, Transition, Qualitative study, Interviews, Grounded Theory, Charmaz

## Abstract

**Background:**

There is a lack of theoretical framework supporting stem cell transplant nurses in their assessment, judgment and caring interventions of sibling stem cell donors.

**Objective:**

The purpose of this study was to explore sibling stem cell donors’ main concerns and how they deal with them before and after donation.

**Method:**

Ten healthy sibling donors, 5 men and 5 women, with a median age of 54 years were included in this study when they were due to donate stem cells to a brother or sister. Data were collected prospectively on three occasions (before the donation and three and twelve months after it) through in-depth interviews, which were recorded and transcribed verbatim for analysis by the Grounded Theory method according to Charmaz.

**Results:**

This study describes the efforts of the ten donors to fulfil their duty as a sibling by doing what they considered necessary in order to help. Their efforts were summarised in a process wherein the grounded theory generated three main categories; Prepare, Promote and Preserve. A clear path of transition leading to fulfilment is evident, starting before the donation and continuing for one year afterwards.

**Conclusions:**

Being a sibling stem cell donor means doing what you have to do to fulfil your duty and if possible, saving the life of a seriously ill brother or sister. The relationship between the siblings is strengthened by the donation process. Sibling stem cell donation appears to be about fulfilment and the theoretical framework may support clinicians in their evaluation and support of donors.

## INTRODUCTION

The rationale behind this study is the complete lack of a theoretical framework to support stem cell transplant nurses in their assessment, judgment and caring interventions when caring for sibling stem cell donors. Haematopoietic stem cell donation is firmly established as a successful treatment for haematological malignancies. Every year more than 15 000 allogeneic stem cell transplantations are performed in Europe [1] and around 280 in Sweden. Haematopoietic stem cells are collected either by bone marrow harvest or, more common nowadays, peripheral blood stem cell collection (PBSC). A donor undergoing bone marrow harvest often spends 2-3 days in hospital, while PBSC collection is usually performed over one or two days at the Out-patient transplant clinic. Generally, a donation from one donor results in one transplantation. If a patient is in need of a second transplantation there might be enough stem cells saved from the donation performed for the first transplantation. However, if that is not the case the donor may be asked to make a second donation.

A stem cell donation where a healthy sibling donates a substance of vital importance for the recipient’s health and survival does not leave the donor untouched. The most common transient side effects of bone marrow harvest are fatigue, lower back pain, pain at the site where the needles were inserted and nausea from the sedation [2-4]. Common transient side effects of PBSC collection are bone pain and headache caused by the injections of Granulocyte Colony-stimulating factor (G-CSF). The donors usually inject themselves with G-CSF at home, 4-5 days prior to donation. Other side-effects that sometimes occur are cramps in fingers, lips and toes due to hypocalcaemia caused by the anticoagulants used during the donation process [5]. Major complications after donating stem cells are rare, but fatalities and life-threatening events such as deep vein thrombosis, splenic rupture and cardiac arrest have been reported [6, 7]. Stem cell donation may enable a complete recovery for the recipient, with a one year survival rate of 70-80%. Although stem cell transplantation offers a potential cure for patients with a variety of diseases, there is a significant risk of acute complications, late side effects and mortality [8, 9]. Despite the lack of studies on the psychosocial consequences for and experiences of adult sibling donors, we know from previous research that these donors are in a vulnerable situation. Negative experiences such as anxiety, pain and guilt, as well as positive experiences including happiness about being a match, an increased sense of self-worth and pride, in addition to a closer relationship with the sick sibling have been described [10, 11].

Sibling donors mostly recover from the donation within 1-2 weeks and experience good physical health [6]. They exist in a social context with a number of close relatives who might be involved in or affected by the donation. Their life situation is most certainly affected by the donation and a theoretical framework of the sibling donor process would be highly useful for enabling healthcare professionals to provide optimum support. Therefore, the aim of this study was to explore sibling stem cell donors’ main concerns and how they deal with them before and after donation in order to develop a theoretical framework.

## MATERIALS AND METHODS

We chose to work inductively using Grounded Theory (GT) according to Charmaz [12]. The focus of this study was sibling donors’ main concerns from the pre-donation evaluation to one year after donation. The constructivist approach enabled us to theorise on the informants’ interpretation that emerged during the interviews. At the same time our ontological assumptions that human beings are social and interactive by nature, and that their actions are driven by their motives [13] were confirmed. Our pre-understanding stems from long experience of caring for stem cell donors and recipients, as well as solid organ donors and recipients. During the whole process we strived to be open to the informants’ main concerns. The constructivist approach resulted in a deeper understanding of when, to what extent and how the concerns occurred and were dealt with during the first year after stem cell donation.

### Ethical Considerations in Research

The study was carried out in accordance with the existing requirements in relation to research on human subjects as set out in the Declaration of Helsinki [14]. Approval was granted by the Regional Ethical Review Board for Southern Sweden (Dnr 2009/655). As there was a risk of pain, distress or violation of integrity, a social worker was assigned to provide counselling if necessary. The informants took part voluntarily and were not in a position of dependence on the researcher. There were no financial incentives. All informants gave their written informed consent.

### Selection and Recruitment

Inclusion criteria were donors who were due to donate stem cells to a brother or sister, with the age for both donor and recipient at least 18 years and ability to speak and understand Swedish. The informants were consecutively recruited from one transplant centre in Sweden with a long history of performing stem cell transplantations. The donors were approached by the first author (AK) at the time of their scheduled evaluation at the Out-patient transplant clinic. True theoretical selection was not employed as we decided to prospectively follow the ten informants initially included. However, we ensured that the sample included sibling relationships of varying closeness to reflect the clinical reality as well as different age groups and genders.

A total of 10 adult stem cell donors, 5 men and 5 women, with a median age of 54 years (range 26-66 years) were included in the study between March, 2011 and December, 2012 (Table **1**). All ten donors who were informed about and invited to take part in the study agreed to participate. They were allowed to decide the time and place for the interviews. No informants were excluded or declined participation after the initial contact. When written consent had been obtained the first interview was scheduled at a location chosen by the informant.

### Data Collection

Data were collected by means of face-to-face interviews. The informants were asked to recall thoughts, emotions and events from the time they were accepted for donation, to the actual donation and onwards during the first year after donation. In total, 29 interviews were performed: before donation (on average 4.5 days prior to donation), and three and twelve months after it. One informant chose not to take part in the last interview. All interviews were conducted by the first author (AK) who is a clinical nurse specialist at the Transplant Centre and has significant professional experience with stem cell donors, but was not involved in the care of the participants in this study. Open-ended questions were used and all interviews started with an open question: “Can you please tell me what it was like when you became aware that your sibling needed a stem cell donor for transplantation and you were asked if you were willing to be tested to become that donor?” “Can you please tell me now, 3 months/one year after the donation, what being the stem cell donor to your sister/brother was like?” The interviews continued with further questions in order to encourage the informants to expand their answers and clarify their thoughts and experiences, including those expressed in previous interviews. The interviews lasted for an average of 60 minutes (range 20 - 198 minutes) and were recorded with a digital voice recorder. The recordings were transcribed verbatim after each interview.

### Data Analysis

In accordance with the recommendations of Hallberg [15] and Glaser [16], we first established whether prospective studies with a GT approach and a similar aim had been previously performed within this particular context. No such study was found. Analysis and data collection were conducted simultaneously. Line by line coding was performed after each interview with focus on actions and processes indicating important categories, qualities or contexts related to the research questions [12]. During the pre-donation interviews detailed memos were written, including reflections that emerged during the analysis and coding process. Later in the process after the 3-month interviews the memos were more theoretically focused as a theory started to emerge. When concluding the data collection at one year after donation the theory that had emerged was confirmed by the last five interviews. We considered that theoretical saturation had been achieved as no new sub or main categories emerged from the data. In line with the constructivism of Charmaz [12], the categories and theory were developed from the patterns revealed by the researchers’ theoretical constructions of the informants’ subjective experiences. In the final step we explored the applicability of the theory in the Out-patient clinic by observing three potential sibling donors in their decision-making process when volunteering for a second donation. One of these donors had already made a second donation. The theory was confirmed and enhanced our understanding of the potential donors’ actions and decisions.

## RESULTS

**Fig. (1):**
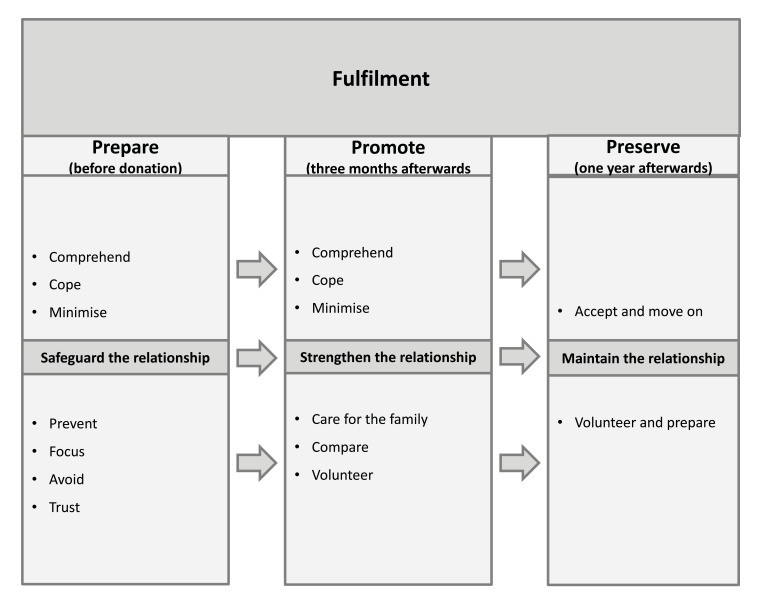
The grounded theory of fulfilment among sibling stem cell donors involving three distinct phases, before donation, three months afterwards and 12 months afterwards.

The core category Fulfilment summarises a process whereby the generated grounded theory contains the three main categories, namely, *Prepare*, *Promote* and *Preserve*. Fulfilment is defined as experiencing a duty to “you do what you have to do” in order to try to save your sibling’s life. This process makes evident a clear path of fulfilment, where the donors do everything in their power to help out, starting pre-donation and continuing until one year after donation. Additionally, the main categories contain several conceptual sub-categories revealing the strategies used to fulfil one’s duty. The outline of the results is presented in (Fig. **1**).

Fulfilment is defined as the experienced duty as a sibling by means of “you do what you have to do” in order to try to save the sibling’s life. Some concepts are the same during the process of change, but the meaning is different as a result of the transition during the first post donation year.

Preparation means a prerequisite for fulfilment of one’s duty to donate. Every effort taken in this phase is aimed at safeguarding the relationship to the sick sibling and to enable the donation.

Promoting means acting in a way that secure the sibling relationship and to support the recipient’s recovery and well-being.

Preservation means maintaining the sibling relationship regardless of closeness or quality.

The common denominator for all the informants was their efforts to minimise the importance of their achievement and instead focus on the relationship with their brother or sister. All informants agreed that donation was about doing what you have to do to the utmost in order to help out in a life threatening situation. It was equally important to the sibling donors to fulfil their duty before and after donation.

Safeguarding the relationship with the sick sibling was the most important strategy throughout the fulfilment process, regardless of the quality of the relationship. All the informants considered that their relationship with the recipient had changed in some way in the year following donation and wished to preserve the changed relationship. Staying in contact mattered more one year after donation, when they opted to spend more time with the recipient than was the case before donation became an issue. The grounded theory approach revealed that the informants’ driving ambition was to fulfil their duty as a human being and as a sibling by donating stem cells in order to save the life of a brother or sister. Regardless of the previous quality of the relationship it was important that it did not become worse.

“*Of course I’ll be there. It’s what you do. Because, I think, it is clear, if you can help a person to live for a few more years, you do*!” (Female donor, 57 years)

Each of the three main categories contains various conceptual sub-categories that explain the different strategies used in the process, i.e., the empirical phenomenon under investigation. In the following, the main categories and sub-categories will be presented in bold italics.

### **Prepare** (pre-donation)

Preparation is a prerequisite for fulfilment of one’s duty to donate. Every effort taken in this phase is aimed at enabling donation. The interviews related to the pre-donation phase took place during the week before the scheduled donation. They tried to ***comprehend*** what was going to happen by seeking information from various sources, searching for vicarious experiences, talking to the sick sibling, discussing the matter with physicians and trying to gain some sort of control in an unfamiliar and frightening situation.

The uncertainty gave rise to a need to ***cope***. Coping strategies involved keeping one’s fingers crossed, adhering to the plan, actively deciding not to worry, relying on destiny, distancing oneself and keeping as calm as possible under the circumstances.

*“I only see the goal that he will recover as quickly as possible”* (Male donor, 32 years)

A common approach among all informants was to ***minimise***, where they actively reduced the importance of their effort and what they were about to do, compared to what the recipient was going through.

*“It’s quite difficult to know what she’s going through when I’m not doing anything special so to speak. I’ll only donate blood and stem cells, and have some injections, but it’s nothing at all compared to her, so for me the treatment is not difficult at all compared to her, who has a really tough time.”* (Female donor, 30 years)

Not all the sibling relationships were described as close and warm, but despite that they all tried to ***safeguard the relationship*** in order to enable the donation and a favourable outcome. Informants who had previously had little contact with the recipient hoped for closeness, although one informant maintained the poor relationship. In order to achieve closeness and prevent possible conflicts they avoided conversations about serious matters that could cause worry or agitation with both the sibling and the rest of the family. As they avoided discussing serious matters with their sibling they speculated about her/his feelings and tried to be positive by encouraging her/him to fight and keep going.

“*I think it will be fine. I hope she doesn’t feel guilty or anything like that, I definitely don’t want her to feel … I just want her to be alive. That’s what I want and I don’t give a damn about anything else*!” (Female donor, 26 years)

A part of the preparation was to ***prevent*** complications or obstacles. They avoided people with infections or took sick leave.

*“That I’m just going to remain healthy. I should have been skiing, but I have cancelled it. And travel down to France and go skiing… I do not risk anything now, that’s how it is. So that it’s going to be as good as possible. It’s just like that.* (Male donor, 66 years)

The donors tried to ***focus*** on the challenge ahead. One way of focusing was by communicating with friends and persons outside their family. They kept up their spirits by adopting a positive attitude, feeling important, hoping for the best outcome and preparing themselves mentally and physically.

*“Right now my body is in order, which I hope will hold until I donate and for me to recover afterwards…there’s nothing that makes me hesitate. This is going to be done, it’s just like that.”* (Female donor, 46 years)

In order to prevent anything negative influencing the donation they tried to ***avoid*** information regarding the prognosis for themselves, as well as the consequences and possible outcome for the recipient. Dealing with uncertainty as best they could on their own served as a protection against doubts and regrets. Finally, a strategy during preparation was to ***trust*** the healthcare professionals’ competence and ability to enable the donor to do what she/he had to do.

### **Promote** (Three Months Post-Donation)

Promoting means acting in a way that strengthens the sibling relationship and supports the recipient’s recovery and well-being. If the recipient’s outcome is not favorable and the relationship is not strong enough the fulfilment of duty is at risk. When interviewed after three months the informants were busy trying to ***comprehend*** and make sense of what had actually happened during the first three months after donation. Pre-donation they had had to cope with the uncertainty about the procedure, while at three months they had to ***cope*** with the uncertainty about the outcome of the recipient’s treatment. Along with hoping they tried to be patient, as they knew that the process took time. Adopting a positive approach was a common strategy in the effort to promote a good outcome. During this phase they also chose to ***minimise*** their effort by playing down both their symptoms and the importance of their intervention. Thus, these three strategies used in the promotion phase were the same as those in the preparation phase but the meaning and the goal of the strategies varied, illustrating a process of change from pre-donation to three months afterwards.

*“There might be a focus on the donor as a hero in some way, or a heroine, and that’s not what it is and it is not like that at all. Instead it is you (healthcare professionals) who work with this and then the patients, who are the real heroes.”* (Male donor, 66 years)

The most essential strategy three months post-donation was ***to strengthen the relationship*** with the recipient. This relationship was fragile for some donors and they were cautious in the contact with the recipient. They prioritised being present at her/his bedside and creating a connection in various ways. If the relationship was good they maintained this situation by putting the sibling first in everything, but even in cases where the relationship was a bit more distanced all informants worried about the recipient and stayed informed about her/his condition.

“*It’s something that makes you closer. We have never been far apart in that sense; it’s more of a geographic distance. Well, we have always been relatively close, but this is something very special*.” (Male donor, 57 years)

When not at the recipient’s bedside the informants engaged in ***family caring***, by means of spending time with their family. They re-connected with family and friends as well as providing information to parents, other siblings and friends who were interested in the donation. They simply felt in charge of providing information to the social network.

*“I have not exactly taken over his role but I am doing a larger share of the other stuff, with family and relatives, where I have assumed responsibility.”* (Male donor, 32 years)

When recovering from the donation they started to ***compare*** their condition and intervention with other similar situations, e.g., kidney donation and persons with chronic conditions such as diabetes. They considered themselves better off than those in the above-mentioned situations. Finally, they learned to accept that the outcome might not be as good as expected.

*“But in this case, it’s like donating blood, that’s how I feel. And, of course, you own your body, but on the other hand, blood is something that is generated again, and everything in the blood, cells and other things will come back. It’s not like giving away a kidney or something.”* (Male donor, 57 years)

In one case, there was a need for a new donation and when this happened the donor ***volunteered*** for a second round without hesitation. She was still focused on doing her utmost and helping out in order to fulfil the duty of saving the sibling’s life. Even donors who were not asked for a second donation expressed their readiness to help and donate again if necessary. Another donor suffered a severe loss when the recipient died shortly before the three month follow up. She thought a great deal about the efforts she had made, what she had experienced and the fact that she had done all she could to save her sibling. At this stage she tried to make plans to move on because she had become quite isolated from the other family members.

### **Preserve** (One Year Post-Donation)

Preservation means maintaining the sibling relationship regardless of closeness or quality. For those with a strong bond it was important to preserve this closeness in order to experience fulfilment. However, the sense of fulfilment was present even for those with a less close relationship. They had still fulfilled their duty but accepted things as they were and moved on. When the donors reflected on the period from three months post-donation to one year afterwards when the interview took place they had no regrets regarding the donation and experienced that their duty was more or less fulfilled. They started to ***accept*** and move on which was evident in the way that they adopted a philosophical approach and acknowledged their own efforts for the first time without playing down or minimising them. Some of the informants distanced themselves from the donation process and took things as they came. In sibling relationships where there was no reunion or closeness the donor adopted a sort of “live and let live” approach.

*“Now I think it’s very much in the background. It actually feels like it was a long time ago. And I do not think about it anymore. In the beginning, I felt very responsible for how it would turn out for him. I don’t feel that way anymore. I have accepted that what happens will happen.”* (Female donor, 62 years)

Donors who had less closeness in their relationship with the recipient nevertheless worked to ***maintain the relationship*** by thinking a great deal about the recipient and trying to accept the relationship as it was. Those with a deep and sincere relationship with the recipient also worked hard to maintain it. Even though they expressed that they had accepted and moved on they constantly thought about the sick sibling and focused on her/his health, which did not only concern keeping in contact by phone or e-mail, but spending time together with her/him. Routines were developed that involved frequent contact. After one year the empathy and closeness had become greater, with the donor acting in a loyal and protective manner. She/he provided support, consolation and inspiration to the recipient and there were many reflections on the quality of the relationship.

“*So it’s obvious, the contact between us has become more frequent, even closer in a way, yes that is what has happened*.” (Male donor, 32 years).

Unfortunately one recipient had a poor outcome after one year, which led to the question of a second donation. The donor whom it concerned again ***volunteered*** for a second donation and started to ***prepare*** herself. This situation evoked a great deal of curiosity about what was actually going on in the recipient’s body. After one year all donors were focused on the outcome, where most of them could relish the fact that their mission was accomplished, while one informant had to start all over again with preparations for a new donation. For her the loop was closed. In the case where the recipient died shortly before the three-month follow-up, the donor had no regrets and considered volunteering to the Swedish registry of unrelated voluntary stem cell donors and donating to an unknown person if possible.

*“Of course, I really would have donated again if that is what she needed. I would have liked that. It is evident that I will always be available for her if needed.”* (Female donor, 26 years)

## DISCUSSION

### Methodological Considerations

To our knowledge this is the first prospective grounded theory study of sibling stem cell donors. We opted to follow Charmaz’s [12] evaluation criteria for rigour in GT studies. Credibility was achieved by methodological accuracy where we searched for words or phrases indicating important categories, qualities or contexts related to the research questions by performing line-by-line coding. At this stage we also wrote memos for each interview, including reflections that emerged during the analysis and coding processes. The third step involved focused coding and in this phase the main concerns were illuminated. Subsequently, theoretical coding was employed to specify the relationships between the codes generated from the focused coding. We used the constant comparative method (CCM) simultaneously on data, codes and categories. In line with the constructivism of Charmaz [12], the categories and theory were developed from the patterns revealed by the researchers’ theoretical constructions of the informants’ subjective experiences.

Finally, credibility was achieved by including elucidative and descriptive quotations in each main category. Quotations from the informants support the principle that the theory is based on the coding process. Originality was ensured by the fact that the study prospectively describes the basic sibling donor process during the first year after donation. Resonance is evident in the GT, while the main categories illustrate the richness of the informants’ experiences during the donation process. The study is valuable, as the findings increase knowledge of the social processes and various choices available to sibling donors during the donation process, thus enabling the transplant nursing profession to provide targeted and person centred support. This study both generates a new hypothesis and confirms established clinical knowledge. The importance of the sibling relationship should not be underestimated. The study also goes one step further by clearly identifying the main concerns during the donation process and how the donors master them.

In order to support the developed theory and strengthen the findings we have inserted quotations from the participants under each category. We have continuously checked the developed concepts and the theory against the data in order to confirm and optimise the result. The theory is probably relevant to the informants in this study and to sibling stem cell donors in developed countries with health care systems similar to those in Scandinavia. After further testing the Grounded Theory of this study might also be applicable within the area of sibling kidney donation. Our assumption is that the aim of fulfilment is generic in a basic human sense and therefore the theory is transferrable to a number of similar contexts.

There are several limitations that need to be noted. First, the study was performed in a Swedish context with only Swedish speaking donors, which limits transferability. Second, the number of participants is small, although the sample is representative of the Swedish donor pool and includes an equal proportion of males and females. We prioritised the unique prospective design by following the ten informants during their first year post-donation, before being completely true to GT methodology in terms of sampling until theoretical saturation occurred. In view of the prospective design and the total of 29 interviews we argue that an in-depth understanding of the donation process was achieved. Third, to some extent we failed to gather experiences from different age groups as the median age is 54 years (range 26-66 years).

#### Reflection on the Findings

To the best of our knowledge, this is the first attempt to use the grounded theory method to understand the core of sibling stem cell donation. The Grounded Theory of Fulfilment has generated a new understanding of the process of stem cell donation to a sibling. The main categories constitute new condensed concepts of how the informants mastered the process of fulfilling their duty both as a human being and a sibling by simply doing what they believed they had to do in order to help a close relative to survive. The clinical implication of this theory is a more specific understanding of the strategies used by sibling stem cell donors to safeguard, strengthen and maintain the relationship with their sick sibling. This understanding reveals that the donors not only do their utmost to help, but also focus on the recipient’s future and hope that she/he will enjoy a reasonably healthy life while constantly minimising their own importance.

The donors were impressively humble in their approach to donation, constantly minimising their role as well as their symptoms during recovery, in addition to making every possible effort to promote the recipients’ health. Our understanding is that this humility serves as an incentive to fulfil their duty. Doing something heroic and then boasting about it seems inappropriate in the given circumstances. Humility in the form of playing down one’s importance and minimising might be a prerequisite for achieving fulfilment and should be acknowledged by tissue transplant professionals. However, as the basic human need for confirmation is still present and the suffering involved in donation, e.g., pain and fear of the outcome, should not be underestimated, confirmation and support are vital.

The social process of change in the relationship with the sick sibling was quite evident. The donors moved from a strong need to safeguard the relationship pre-donation, to strengthening it due to their happiness that the donation had contributed to increased closeness in most but not all cases. After one year they wished to maintain this closeness, which they perceived as highly valuable, regardless of the outcome of the donation. Although we already knew that the sibling relationship is important [10, 11, 17], our grounded theory revealed that it constitutes an important part of the desire for fulfilment. This is a completely new and clinically important understanding when evaluating possible donors. Even among siblings who described their relationship as distanced it was obvious that being able to help during a limited period of their life was an important driving force for personal fulfilment.

The strategies for dealing with the main concerns in each phase of the process were reasonable and relevant. During preparation we argue that it is important to support the donors’ efforts to prepare themselves and not trigger too many doubts, as in our study they were highly focused on and motivated for the donation. A person centred approach involves being sensitive to their motives and answering their specific questions based on their individual needs, concerns and preferences. Interestingly, the will to volunteer for a second donation was evident during the first three months post donation, suggesting a strong desire to promote a favourable outcome. However, fulfilment was disrupted by an unfavourable outcome of the donation. In our clinical practice we have observed this readiness even earlier than three months post donation. During the promotion phase the donors compared their effort with other living donation procedures, e.g., kidney donation, and minimised their own effort. Previous studies in the field of living kidney donors [18-21] and stem cell donors [11] show the same pattern of minimising as well as a profound sense of loneliness and feeling abandoned by the healthcare professionals. We believe it is unfortunate that clinicians often focus on the survival of the recipient and therefore tend to underestimate the suffering involved in being a healthy person who donates a vital tissue or organ. By paying attention to the inside perspective and adopting our grounded theory as a framework in clinical practice, the encounter with the sibling donor could be enhanced and more relevant support provided.

#### Clinical Implications

A thorough understanding of the social process of fulfilment when donating stem cells to a sibling enables customized and person centered support in every phase of the donation process. The expected outcome of the donation was not necessarily the development of more closeness, as the expectations regarding closeness were fairly realistic. Instead, the circumstances provided an opportunity for the donors to do what they believed they had to do. Thus, the opportunity of doing what you believe you have to do is of far greater importance than the potential improvement in the sibling relationship. It is not the quality of the sibling relationship that is decisive for the way the donors manage the donation process, but how they view their possibility of helping out. As a consequence, during the donor evaluation we should try to ascertain that the key motive of fulfilment exists rather than try to judge whether the relationship is close enough. This knowledge enables a targeted discussion during the evaluation in order to identify siblings who lack the core incentive for donation, which could mean the absence of the necessary motivation to proceed with the donation. This grounded theory constitutes a framework for long-term follow up, i.e., the first post donation year, which is requested by donors but not always delivered by transplant professionals. It also enriches transplant nursing by ensuring the provision of evidence-based care.

## CONCLUSION

In conclusion, sibling stem cell donation is about doing one’s utmost to help, fulfil one’s duty and preserve the sibling relationship that developed during the donation process. When the mission is accomplished it is possible to accept and move on, while at the same time being aware of the preparations needed for a possible second round. When a recipient died it was still possible to experience fulfilment in the sense that everything possible was done. Our profound understanding is that in order to become a sibling donor it is essential to be aware of one’s duty, where fulfilment is the core incentive. When this incentive is missing it is presumably difficult to find the motivation to prepare for and make the donation. Although this assumption is confirmed in our everyday clinical practice, further studies are needed to verify it from a scientific perspective.

## Figures and Tables

**Table 1:** Demographics and characteristics of the donors.

*Characteristics*	n =10 *n*
*Age, years* Median (range)	54 (26-66)
*Sex* Female Male	5 5
*Occupation* Employed Disability pension	7 3
*Marital status* Married/living together Single	7 3
*Donation method* PBSC BM	9 1
*Gender recipient* Female Male	6 4
*Relationship with recipient* Frequent contact Occasional/no contact	6 4

PBSC = Peripheral Blood Stem Cells

BM= Bone marrow
